# Estimation of Phylogeny Using a General Markov Model

**Published:** 2007-02-25

**Authors:** Vivek Jayaswal, Lars S. Jermiin, John Robinson

**Affiliations:** 1 School of Mathematics and Statistics, University of Sydney, NSW 2006, Australia; Sydney University Biological Informatics and Technology Centre, University of Sydney, NSW 2006, Australia; 2 School of Biological Sciences, University of Sydney, NSW 2006, Australia; Sydney University Biological Informatics and Technology Centre, University of Sydney, NSW 2006, Australia. Unité de Biologie Moléculaire de Gène chez les Extrêmophiles, Institut Pasteur, 75724 Paris Cedex 15, France; 3 School of Mathematics and Statistics, University of Sydney, NSW 2006, Australia

**Keywords:** Phylogenetics, Maximum Likelihood, Reversibility, Tests for Symmetry, Nucleotide Sequence Evolution

## Abstract

The non-homogeneous model of nucleotide substitution proposed by Barry and Hartigan (*Stat Sci*, 2: 191–210) is the most general model of DNA evolution assuming an independent and identical process at each site. We present a computational solution for this model, and use it to analyse two data sets, each violating one or more of the assumptions of stationarity, homogeneity, and reversibility. The log likelihood values returned by programs based on the F84 model (*J Mol Evol*, 29: 170–179), the general time reversible model (*J Mol Evol*, 20: 86–93), and Barry and Hartigan’s model are compared to determine the validity of the assumptions made by the first two models. In addition, we present a method for assessing whether sequences have evolved under reversible conditions and discover that this is not so for the two data sets. Finally, we determine the most likely tree under the three models of DNA evolution and compare these with the one favoured by the tests for symmetry.

## 1 Introduction

The evolutionary relationship between a set of *k* homologous sequences of *N* nucleotides can be represented by a *k*-leaved bifurcating tree where each leaf node represents a known sequence and each internal node represents an ancestral sequence (which is almost always unknown). The phylogeny of the *k* sequences can be inferred by using maximum-likelihood methods, which rely on models of nucleotide substitution to infer the most likely tree. Popular phylogenetic methods, like those implemented in PHYLIP (Felsenstein 2004a), PAUP* (Swofford 2002), and Tree-Puzzle (Schmidt et al 2002), use models of nucleotide substitution that assume the evolutionary process is stationary, homogeneous, and reversible. Although a detailed mathematical description of stationarity, homogeneity and reversibility can be found in Ababneh et al (2006a), we will give a brief description of these terms in the context of molecular phylogenetics. Stationarity implies that the marginal probabilities of the four nucleotides remain constant over all the nodes of a given tree. Homogeneity implies that the instantaneous rate matrix (described in eg, Lanave et al 1984, Kishino and Hasegawa 1989) is constant over an edge (local homogeneity) or constant over the entire tree (global homogeneity). Reversibility implies that the probability of sampling nucleotide *i* from the stationary distribution and going to nucleotide *j* is the same as the probability of sampling nucleotide *j* from the stationary distribution and going to nucleotide *i*, where *i*, *j* = {*A*,*C*,*G*,*T*} (Bryant et al 2004). Reversibility, therefore, implies that the process is stationary and permits us to ignore the direction of evolution. The assumptions of stationarity, homogeneity and reversibility are often violated by the data, resulting in an elevated probability of incorrect phylogenetic results (for examples of the complexity of the problem, see Ho and Jermiin 2004; Jermiin et al 2004).

In a landmark article, Barry and Hartigan (1987a) considered a general Markov model for unrooted trees, the assumptions being that the process relating each pair of nodes in the tree is Markovian and the sites are independent and identically distributed. Their model does not make the assumption of stationarity, homogeneity (local or global) or reversibility, so it is more general than the non-stationary but locally homogeneous models considered by Yang (1994) and Yang and Roberts (1995). Barry and Hartigan (1987a) considered a *k*-taxa tree with 2*k*−3 *Q*-matrices, one for each edge of the tree. Each *Q*-matrix represents the joint probability distribution of nucleotides at the two ends of the associated edge and is a 4 × 4 matrix. Since the sequences at internal nodes are not known, we can only observe the 4*^k^* different combinations of nucleotides at the leaf nodes. These combinations together represent the joint probability distribution of the nucleotides at leaf nodes and can be written as a function of the *Q*-matrices. Thus, the likelihood of the observed sequences is a function of the set of *Q*-matrices and can be maximised by determining the maximum-likelihood estimates of the *Q*-matrices. The algorithm for obtaining the maximum-likelihood estimates was suggested by Barry and Hartigan (1987a) but has not received the attention that other aspects of their paper have, especially calculation of LogDet distance (Lockhart et al 1994; Steel 1994), probably because the large number of parameters was assumed to make the interpretation difficult.

We revisit Barry and Hartigan’s (1987a) model, and describe it and the estimation algorithm in new notation — we also present a program written in Java^™^ to implement it. We examine the information that can be obtained from the estimates by applying their algorithm, henceforth referred to as the BH algorithm, to two sets of data, one comprising mitochondrial DNA from seven hominoids, where there is apparent stationarity and homogeneity, and another comprising 16S ribosomal RNA genes from five bacterial genomes, where problems due to lack of stationarity and homogeneity have been noted previously by Galtier and Guoy (1995) as well as Foster (2004). Further, we compare the results obtained from our program with those obtained using simpler models, ie, the F84 model (Kishino and Hasegawa 1989), implemented in DNAML from the PHYLIP program package (Felsenstein 2004a), and the general time reversible (GTR) model (Lanave et al 1984), implemented in PAUP* (Swofford 2002). A likelihood ratio test (Huelsenbeck and Crandall 1997), based on the log likelihood values obtained using the phylogenetic programs, is used to determine whether one or more of the assumptions of stationarity, homogeneity, and reversibility are violated.

The joint probability distribution values for each edge of the tree can be used to determine (*a*) marginal probabilities at the nodes (internal nodes as well as leaf nodes), and (*b*) the joint probability distribution of a pair of leaf nodes. The assumption of stationarity can be examined by comparing the marginal probabilities at different leaf nodes (Ababneh et al 2006b). Since Barry and Hartigan’s (1987a) method gives estimates of the joint distribution of the two end points of each edge, we can evaluate the hypothesis of reversibility by examining the joint distribution — it should be symmetric if the process is reversible. We do so for the two sets of data mentioned earlier, obtaining the surprising result that the stationary and homogeneous model for the hominoid data appears to be not reversible along some of the edges. Such comparisons seem to be possible for only a part of the tree for the bacterial data since this data set is not stationary.

## 2 A General Markov Model on Trees

The general Markov model for phylogenetic trees proposed by Barry and Hartigan (1987a) will be given using a notation that permits a more compact description. Consider an unrooted binary tree, *T*, (for definitions, see Chapter 1 of Semple and Steel (2003)) with *l* leaves, *l –* 2 internal nodes (or vertices), and 2*l* − 3 edges, for *l* ≥ 0. For convenience, we include *l* = 1 with 0 internal nodes and 0 edges. Denote leaves by *L* = {−1, …, −*l*} and internal nodes by *I* = {1, …, l − 2} (the notation of positives and negatives derives from the merge matrix given by the hierarchical clustering algorithm, *hclust*, in the S-PLUS or R packages). The set of all vertices is *V* = *L* ∪ *I*. Denote edges by *E* = {(*i*, *j*): *i*,*j*∈*V* and adjacent}. By inserting a node numbered 0, called a root node, on any edge, and thus increasing the number of nodes and the number of edges by one, the unrooted tree can be converted into a rooted binary tree. If an edge (*i*, *j*) of the unrooted tree is deleted then two rooted sub-trees *T*_(_*_i_*_,_*_j_*_)_ and *T*_(_*_j_*_,_*_i_*_)_ are formed with roots at *i* and *j*, respectively.

The tree will be used to describe a model for evolutionary relationships at a site in the DNA, as in Barry and Hartigan (1987a), by considering the joint distribution of the four bases *B* = {*A*,*C*,*G*,*T*} at the leaves. First consider the joint distribution at ends of any edge. Let *X_i_* and *X_j_* be the values taken by bases at nodes *i* and *j* of the edge (*i*, *j*). Write

(1) $$Q_{\left(i,j\right)}(x,y)=P(X_{i}=x,X_{j}=y)$$

for *x, y* ∈ *B*, as the joint probability. Note that, since consistency of marginal distributions at internal nodes is required, for *i* ∈ *I*,

$$P(X_{i}=x)=Q_{i}(x)=\sum_{y\in B}Q_{\left(i,j\right)}(x,y)$$

for any *j* such that (*i*, *j*) is an edge.

More generally, let **X** = (**X***_L_*, **X***_I_*) denote the vector of random variables with **X***_L_* = (*X*_−1_, …, X_−l_) and **X***_I_* = (*X*_1_,…,*X_l_*_−2_), and with each *Xi* taking values in *B.* Let *Q_T_* (**x**) = *P*(**X** = **x**) be the joint distribution of the bases at the nodes of *T*. The joint distribution of the bases at the leaves is then

(2) $$Q_{L}(x_{L})=\sum_{x_{i}:i\in I}Q_{T}(x).$$

Further, if *L*_(_*_i_*_,_ *_j_*_)_ = *L* ∩ *T*_(_*_i_*_,_ *_j_*_)_ and *I*_(_*_i_*_,_ *_j_*_)_ = *I* ∩ *T*_(_*_i_*_,_ *_j_*_)_ denote the sets of leaf nodes and internal nodes, respectively, in *T*_(_*_i_*_,_ *_j_*_)_, then **X**_(_*_i_*_,_ *_j_*_)_ and **X**_T(_*_i_*_,_ *_j_*_)_ denote the vectors of the values of bases in *L*_(_*_i_*_,_ *_j_*_)_ and *T*_(_*_i_*_,_ *_j_*_)_, respectively, and

(3) $$Q_{L_{\left(i,j\right)}}\left(x_{L_{\left(i,j\right)}}\right)=\sum_{x_{k}:k\in I_{\left(i,j\right)}}Q_{T_{\left(i,j\right)}}\left(x_{T_{\left(i,j\right)}}\right)$$

Take the model to be Markovian, so that, given (*X_i_*, *X_j_*) = (*x_i_*, *x_j_*), the conditional distribution of the bases on the leaves of the rooted sub-trees *T*_(_*_i_*_,_ *_j_*_)_ and *T*_(_*_j_*_,_ *_i_*_)_, given by deleting edge (*i*, *j*), are independent. Under this Markovian model the joint distribution *Q_L_*(**x***_L_*) can be written as a product of terms involving only *Q*_(_*_i_*_,_ *_j_*_)_(*x_i_*, *x_j_*) and *Q_i_*(*x_i_*) for all edges (*i*, *j*) and all nodes *i*. At each site α = 1, …, *N* the value of a base at the *i*-th leaf, *x_iα_* is known, but at internal nodes the base can take any value in *B*. Let *B_iα_* = {*x_iα_*} if *i* ∈ *L*, and *B* if *i* ∈ *I*. Then the joint probability distribution of leaf nodes at site α can be expressed as

(4) $$Q_{L,\alpha }(x_{L})=\sum_{x_{i}\in B}\sum_{x_{j}\in B}I(x_{i}\in B_{i\alpha },x_{j}\in B_{j\alpha })$$

$$Q_{\left(i,j\right)}\left(x_{i},x_{j}\right)P\left(L_{\left(i,j\right)}|x_{i}\right)P\left(L_{\left(j,i\right)}|x_{j}\right).$$

where *I*(*x_i_* ∈ *B_iα_, x_j_* ∈ *B_jα_*) is an indicator function that takes the value 1 if both *x_i_* and *x_j_* represent leaf nodes, and 0 otherwise. Also,

$$P\left(L_{\left(i,j\right)}|x_{i}\right)=\frac{Q_{L_{\left(i,j\right)}\cup \{x_{i}\}}\left(x_{L_{\left(i,j\right)}},x_{i}\right)}{Q_{i}(x_{i})}$$

This formula can be applied recursively to the joint distribution on a smaller tree,

$$Q_{L_{\left(i,j\right)}}\left(x_{L_{\left(i,j\right)}}\right)$$

until trees with only one edge are reached.

Notice that it is not necessary in this general case to put any restrictions on the model producing the joint distributions on each edge, other than consistency at internal nodes noted earlier.

## 3 Estimation

For an unrooted binary tree, *T*, based on *k* homologous sequences, each having *N* sites, Barry and Hartigan (1987a) gave a method of estimating the set of *Q*_(_*_i_*_,_ *_j_*_)_(*x*, *y*) for *x*, *y* ∈ *B*, (*i*, *j*) ∈ *E* by maximizing the log likelihood of the bases at the leaves. Using (4), the log likelihood for an unrooted tree is

(5) $$\begin{array}{l} L=\sum_{\alpha =1}^{N}\text{log}Q_{L,\alpha }(X_{L})\\ =\sum_{\alpha =1}^{N}\text{log}\sum_{x\in B}\sum_{y\in B}I\left(x\in B_{i\alpha },y\in B_{j\alpha }\right)\\ Q_{\left(i,j\right)}(x,y)P\left(L_{\left(i,j\right)}|x\right)P\left(L_{\left(j,i\right)}|y\right) \end{array}$$

Now, maximizing *L* with respect to *Q*_(_*_i_*_,_ *_j_*_)_(*x*, *y*) subject to

$$\sum_{x,y\in B}Q_{\left(i,j\right)}(x,y)=1$$

requires equating the derivatives of *L* + λ (∑*_x_*_,_ *_y_*_∈_ *_B_* *Q*_(_*_i_*_,_ *_j_*_)_(*x*, *y*) − 1) with respect to *Q*_(_*_i_*_,_ *_j_*_)_(*x*, *y*) and λ to zero, which leads to the updating equation

(6) Q(i,j)(x,y)=1N∑α=1NI(x∈Biα,y∈Bjα)Q(i,j)(x,y)P(L(i,j)|x)P(L(j,i)|y)QL(xL).

In order to minimize computational time, suitable initial values are chosen for all *Q*_(_*_i_*_,_*_j_*_)_(*x*, *y*). Then (6) is used repeatedly on all edges to update the left hand side using current values for the right hand side until the process converges. Call the values obtained

$${\hat{Q}}_{\left(i,j\right)}(x,y).$$

### 3.1 Precise Fit At Leaf Nodes

If *i* ∈ *L*, then summing in (6) over *y* ∈ *B* gives

$${\hat{Q}}_{i}(x)=\frac{N_{i}(x)}{N}$$

where *N_i_*(*x*) denotes the number of sites at leaf *i* that have base *x*. Thus the maximization leads to a precise fit at the leaves.

### 3.2 Internal Consistency

If *i* ∈ *I* and edges (*i*, *j*) and (*i*, *k*) are in *E*, then, if the sum over *y* in *Q̂* _(_*_i, j_*_)_ (*x, y*), and over *z* in *Q̂* _(_*_i,k,_*_)_ (*x*,*z*), are equal, these estimates of the marginal probabilities at internal nodes are consistent. Now from (6) we get

$$\begin{array}{l} \sum_{y\in B}{\hat{Q}}_{\left(i,j\right)}(x,y)=\frac{1}{N}\sum_{\alpha =1}^{N}\frac{\Sigma_{y\in B}I\left(x\in B_{i\alpha },y\in B_{j\alpha }\right){\hat{Q}}_{\left(i,j\right)}(x,y)P\left(L_{\left(i,j\right)}|x\right)P\left(L_{\left(j,i\right)}|y\right)}{{\hat{Q}}_{L}(x_{L})}\\ =\frac{1}{N}\sum_{\alpha =1}^{N}\frac{{\hat{Q}}_{L\cup \{i\}}(x_{L\alpha },x)}{{\hat{Q}}_{L}(x_{L\alpha })} \end{array}$$

where **x***_Lα_* is the vector of values of bases of leaves at the αth site. The same formula is obtained by summing over *z* in

$${\hat{Q}}_{\left(i,k\right)}(x,z)$$

showing that the estimates are internally consistent.

## 4 Algorithm Implementation

The BH algorithm was implemented in Java (Java*^™^* 2 Platform Standard Edition, Version 1.4.2_03) using an object-oriented approach. The main classes in the program are NewickTreeTraversal, BranchDetails and MaximumAverageLikelihood. The class NewickTreeTraversal reads the unrooted tree in Newick format (Felsenstein 2004b) and constructs a binary tree. Each node is linked to a maximum of three nodes ie one parent node and two descendant nodes. The class BranchDetails stores the joint probability distribution values along each edge of the binary tree. The class MaximumAverage Likelihood makes use of the above-mentioned classes to compute the log likelihood values and update joint probability distribution values (using formulae described in Section 3) for a user-specified tree. It also generates an output file containing the final joint probability distribution values along each edge and the log likelihood value for the entire tree. The joint probability distribution values can be used to compute divergence matrices — a helper program has been written in Java*^™^* for this purpose.

We make use of recursion to compute the joint conditional probability distribution of all the leaf nodes connected to the sub-tree rooted at node *i*, ie *P*(*L*_(_*_i_*_,_ *_j_*_)_*|x*). The method starts by calculating the joint probability of node *i* and its immediate descendant nodes. If node *i* is an internal node, *x* ∈ *B* and the joint probability distribution is the sum of joint probability values obtained for different nucleotide values at node *i*. If a descendant node is an internal node, we consider the sub-tree rooted at the descendant node and compute *P*(*L*_(_*_i_*_,_*_j_*_)_*|x*). This process is repeated until the leaf nodes are reached.

The initial joint probability distribution values (ie, the *Q*-values) along the edges of the binary tree are provided by the user. At the end of each iteration, we compare the *Q*-values before and after updation. If the sum of the square of differences is greater than the user-specified value, the *Q*-values are updated and the next iteration begins. If none of the edges need to be updated, it implies that convergence has been achieved, and the program terminates.

To improve the program’s performance, henceforth referred to as the BH program, for a given data set of matched nucleotide sequences, all unique patterns are identified at the beginning of the program. The log likelihood value is computed only once for each unique pattern and the result is multiplied by the number of times a particular pattern occurs — this is a commonly used procedure to reduce the time needed to estimate the likelihood of a tree.

The software will be available for download from http://www.usyd.edu.au/SUBIT/.

### 4.1 Computation of Edge Length

If the nucleotide sites are independent and identically distributed and the underlying model of evolution is stationary, homogeneous and reversible, we can compute edge lengths (Lanave et al 1984; Tavaré 1986; Rodríguez et al 1990) using the formula

$$\delta_{ij}=-t\sum_{h=1}^{4}\pi_{h}r_{hh}$$

where δ*_ij_* denotes the distance between sequences at nodes *i* and *j* in terms of expected number of substitutions per site, *t* denotes time, π*_h_* denotes the *h-*th element of the diagonal matrix of stationary probabilities, and *r_hh_* denotes the *h-*th diagonal element of the rate matrix. A method for determining asynchronous distances was proposed by Barry and Hartigan (1987b). Although their method can be applied to the general model, if the marginal probabilities at the two ends of an edge are different, the distances are asymmetric (ie, for an edge (*i*, *j*), the distance from *i* to *j* and from *j* to *i* are different). In our paper, we have averaged the distances over the two possible directions of traversal for the purpose of edge length comparison with DNAML. Since the BH algorithm is based on joint probability distributions along the edges and does not require branch length optimization, the averaging of branch lengths does not affect the maximum-likelihood computation.

### 4.2 Variation in log likelihood values

For a given data set of homologous nucleotide sequences, the log likelihood value at convergence depends on the initial set of *Q*-values. This was observed in both five-taxa and seven-taxa trees irrespective of the tree selected. This indicates the presence of multiple local maxima on the likelihood surface even for the most likely tree. This is an important result because former studies of the problem of multiple maxima on the log likelihood surface have assumed stationary, homogeneous, and reversible models of evolution. Chor et al (2000) showed that even for simple models of evolution, multiple maxima are possible while Rogers and Swofford (1999) used simulation to show that the best tree is unlikely to have multiple maxima.

For the two data sets analysed below, convergence to a local maximum, different from the global maximum, was observed only if the *Q*-values chosen were extreme; for example, a *Q*-matrix with all the joint probabilities being equal or a *Q*-matrix with diagonal elements much smaller than off-diagonal elements. From the *Q*-matrices that converged to the global maximum, we randomly selected one with a value of 1/8 along the main diagonal and 1/24 elsewhere for the computation of log likelihood values mentioned in section 5.

## 5 Application to two sets of homologous sequences

Under the Markovian model of DNA evolution, the process of evolution may or may not be stationary and homogeneous. We consider both cases and argue that the general model of DNA evolution proposed by Barry and Hartigan (1987a) is useful in both cases. For each data set, we (*i*) used three matched-pairs tests of homogeneity (Bowker 1948; Stuart 1955; Ababneh et al 2006b) to determine whether the sequences could be assumed to have evolved under stationary and homogeneous conditions (a prerequisite for using most phylogenetics methods); (*ii*) determined the degrees of freedom needed in order to compare phylogenetic results using likelihood-ratio tests; (*iii*) estimated and compared the trees; and (*iv*) conducted a comparison of edge lengths, divergence matrices and substitutional biases. We show that Barry and Hartigan’s (1987a) method provides a useful reference point for choosing appropriate models of substitution, and the means for assessing whether the evolutionary process is reversible; such a method appears to be unavailable in the current literature.

### 5.1 Hominoid Data

We considered an alignment of 1809 nucleotides from the mitochondrially-encoded NADH dehydrogenase subunit 5 genes of (with abbreviated name and Genbank Accession numbers given in parentheses): Human (Hsap, NC_001807), Chimpanzee (Ptro, NC_001643), Bonobo (Ppan, NC_001644), Gorilla (Ggor, NC_001645), Orangutan (Ppyg, NC_001646), Gibbon (Hlar, NC_002082), and Macaque (Msyl, NC_002764). The three codon sites were separated into different alignments using a program called CODONSPLIT (by IB Jakobsen) before being analysed.

#### 5.1.1 Assessment of phylogenetic assumptions

The alignments of first, second, and third codon sites were examined independently using the matched-pairs tests of symmetry (Bowker 1948), marginal symmetry (Stuart 1955), and internal symmetry (Ababneh et al 2006b). Given that each of these tests involve multiple comparisons of related sequences, it was necessary to interpret the *p*-values with caution. The matched-pairs tests of homogeneity produced *p*-values in the range of 1.000 to 0.024 for the first and second codon sites (Tables 1 and 2), and in the range of 0.996 to 0.006 for the third codon sites (Table 3). For the 21 pairwise comparisons, only 1 *p*-value was observed to be lower than 0.05 for the first and second codon sites whereas approximately one-fourth of the *p*-values for the third codon site were found to be lower than 0.05. These results are consistent with evolution under stationary and homogeneous conditions for first and second codon sites but not for third codon sites. Interestingly, all the low *p*-values observed for third codon sites involved comparisons with Orangutan, indicating real differences.

Given that the alignment of third codon sites provides some evidence against the evolutionary process being stationary and homogeneous, the following phylogenetic analyses were done using an alignment of first and second codon sites only. Assuming stationarity, homogeneity, and reversibility, the GTR model, considered over the entire tree, would be appropriate for inferring the most likely tree. If we constrain the assumptions further by assuming that the six rate parameters in the GTR model can be reduced to two rate parameters (ie transitions and transversions), then the F84 (Kishino and Hasegawa 1989) model would be sufficient to predict the most likely tree. We determined the most likely tree by using DNAML from the PHYLIP program package (Felsenstein 2004a), PAUP* (Swofford 2002), and the BH program.

#### 5.1.2 Calculating the degrees of freedom

In order to compare the fit of the alignment to trees inferred using DNAML (Felsenstein 2004a), PAUP* (Swofford 2002), and the BH program, the degrees of freedom are needed for each estimate. Both DNAML and PAUP* consider a stationary, homogeneous, and reversible process, so a single rate matrix applies to the entire tree, and the degrees of freedom is the sum of the number of edges and the number of parameters in the rate matrix. The F84 model has five parameters in the rate matrix and the GTR model has nine parameters in the rate matrix. However, in order to obtain the edge lengths in terms of the expected number of substitutions per site, the expected rate of substitution is set to 1 (Yang and Roberts 1995), so the number of free parameters in the F84 and GTR models is reduced by one. Accordingly, for a seven taxa tree, the degrees of freedom is 15 for results obtained using the F84 model and 19 for results obtained using the GTR model; the difference in the degrees of freedom for these two models is four. The model proposed by Barry and Hartigan (1987a), which does not assume stationarity, homogeneity or reversibility, has nine degrees of freedom along each edge and three degrees of freedom at each node. Thus, the degrees of freedom for a seven taxa tree inferred using the BH algorithm is 135, and the difference in the degrees of freedom is 116 and 120, respectively, for the GTR and F84 models (in relation to trees inferred using the BH algorithm).

#### 5.1.3 Inferring and comparing the trees

**Figure 1:**
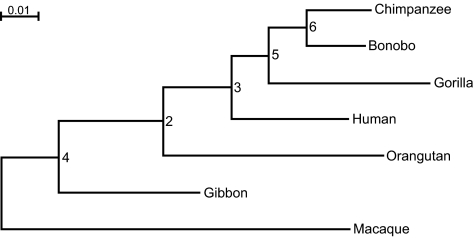
The most likely tree of the hominoids inferred using F84 and GTR models (the bar corresponds to 0.01 substitutions per site).

The most likely tree obtained using DNAML and PAUP* is shown in Figure 1. As the difference in log likelihood (ln*L*) obtained using these two programs is 9.2 (−3635.445 for PAUP* and −3644.645 for DNAML), 2 × ln*L* = 18.4. Under the hypothesis that the GTR model can be reduced to the F84 model for these data, we might expect the difference to be distributed approximately as a chi-squared variate with four degrees of freedom. Thus, for the hominoid data set, there is evidence that the F84 model is not sufficient to explain the evolutionary process.

To determine the most likely tree inferred by the BH program, we performed an exhaustive search of tree-space. The Newick representation of all the 945 possible unrooted binary trees was generated using the TreeGen program (Wolf et al 2000). The BH program used these trees as input to generate the log likelihood value for each tree. In all cases, the *Q*-matrices showed internal consistency and a precise fit at the leaf nodes. The three most likely trees and their log likelihood values are shown in Table 4. The most likely tree inferred by using the BH algorithm is the same as those inferred by DNAML and PAUP*. Interestingly, the second most likely tree in Table 4 is the one that is commonly thought to represent the hominoid evolution (see eg Raaum et al 2005) whereas the third most likely tree in Table 4 is the one inferred by Hudelot et al (2003).

We compared the trees in Table 4 using the SH-test by Shimodaira and Hasegawa (1999) and the approximately unbiased (AU) test by Shimodaira (2002), which are implemented in CONSEL (Shimodaira and Hasegawa 2001). The results in Table 5 show that the two most likely trees are statistically indistinguishable, possibly due to the sequences being too short (1206 bp) to rule out stochastic error, which can interact with systematic errors and prevent identification of the correct tree.

For the most likely tree presented in Table 4, the log likelihood values returned by PAUP* and BH are −3635.445 and −3540.684, respectively, so 2 × ln*L* = 189.5226, which is large compared to a chi-squared distribution with 116 degrees of freedom. Since the large difference in log likelihood cannot be explained by the difference in the degrees of freedom of the two models, the likelihood ratio test suggests that one or more of the three assumptions of stationarity, homogeneity and reversibility are violated by the hominoid data set. Given the results from the matched-pairs tests of symmetry, marginal symmetry and internal symmetry, there is reason to suspect that the assumption of reversibility is violated.

Given that there may be doubt about the accuracy of the asymptotic approximation in cases like this, we verified the results by using parametric bootstrapping. The parameters values for the GTR model were estimated on the most likely tree using the HyPhy program (Kosakovsky Pond et al 2005). One thousand alignments of 1206 nucleotides were generated on the parameter values and the most likely tree using the Seq-Gen program (Rambaut and Grassly 1997). For each alignment, we estimated the log likelihood of the data, given the tree and the GTR model or given the tree and the BH model. The values for 2 × ln*L* ranged from 68.29 to 152.60 with a mean of 109.90 and a median of 109.60. Approximately 71% of the values lay between 116 ± 15.23, where the latter value corresponds to the standard deviation of a χ^2^_116_. This shows that the large difference in log likelihood values returned by PAUP* and BH program for the hominoid data set is significant.

#### 5.1.4 Tree-dependent comparison of edge lengths

The joint probability distribution values returned by BH were used to obtain edge lengths by averaging over the two possible directions of traversal. If the process is stationary, homogeneous, and reversible, the values obtained over the two directions of traversal would be the same. However, the alignment of first and second codon sites of the hominoid data is not consistent with evolution under stationary, homogeneous and reversible conditions, so the averaged edge lengths will only provide a rough estimate of the expected number of substitutions along each edge. Nonetheless, the edge lengths obtained using DNAML and BH are similar (Table 6).

#### 5.1.5 Evaluation of Divergence Matrices and Substitution Biases

Given two neighbouring edges (*i*, *j*) and (*k*, *j*), the joint probability distribution for the pair (*i*, *k*) can be estinated as

$$\sum_{x_{j}=1}^{4}\frac{Q_{\left(i,j\right)}\left(x_{i},x_{j}\right)Q_{\left(j,k\right)}\left(x_{j},x_{k}\right)}{Q_{j}\left(x_{j}\right)}$$

A generalisation of this formula, obtained by summing over all internal nodes in the path from node *l* to node *m*, and multiplying by *N* gives the estimated divergence matrix for any pair (*l*, *m*). For the hominoid data set, the divergence matrices computed using the estimated joint probability distribution values along each edge of the tree are close to the observed divergence matrices. In Table 7 we give the estimated and observed divergence matrix values for the Macaque-Bonobo pair. The values indicate that the general model of DNA evolution approximates the actual process of evolution quite well.

This is typical of the fit of divergence matrices for all pairs of leaf nodes. To compare the differences between observed and estimated divergence matrices within a statistical context, we calculated a chi-squared test statistic using the formula

(7) $$X^{2}=\sum_{i=1}^{4}\sum_{j=1}^{4}\frac{{\left(O_{ij}-E_{ij}\right)}^{2}}{E_{ij}}$$

where *O* denotes the observed divergence matrix, and *E* denotes the estimated divergence matrix. This goodness-of-fit index has values in the range 0.07 (Chimpanzee-Bonobo pair) to 8.19 (Human-Macaque pair). Since the marginal probabilities for each pair of leaf nodes are known, the degrees of freedom for the above-mentioned test statistic cannot exceed nine. We would obtain exactly nine degrees of freedom if *E* was known precisely apart from the marginal probabilities.

The divergence matrices computed in section 5.1.1 were found to be symmetric for all comparisons between the leaf nodes (Tables 1 and 2). However, when we looked at the estimated joint probability distributions for individual edges, we observed a distinct lack of symmetry. We used the Bowker (1948) and Stuart (1955) test statistics using *NQ*(*x*, *y*), for *x*, *y* = 1, …, 4, as pseudo-observations. The resulting pseudo-*p*-values are provided in Table 8. Although these values are based on *Q*-matrices estimated by the BH program and therefore are not the true *p*-values, they are useful indices for measuring symmetry and provide a clear indication of lack of symmetry for internal node to leaf node edges.

An examination of the corresponding estimated joint probabilities shows a bias for *A* → *G* and *C* → *T* substitutions over *G* → *A* and *T* → *C* substitutions for ancestral to leaf node transitions (Table 9). Since a reversible Markov process would result in a symmetric joint distribution of the end points of an edge, there is evidence that the process in fact is not reversible. This observation is consistent with the earlier research on mammalian genes. Wise et al (1998) found a directional bias in nucleotide substitutions in the human mitochondrial genome whereas Eyre-Walker (1999), using a stationary and infinite-sites model, found that a base composition bias in mammals cannot be explained by the mutation bias hypothesis. Subsequently, assuming a stationary model, Smith and Eyre-Walker (2001) found that the synonymous codon bias in humans cannot be explained by the mutation bias hypothesis whereas Galtier et al (2001) have argued that the evolution of GC-content in mammals is explained by a biased gene conversion hypothesis.

### 5.2 Bacterial Data

We analysed a second data set comprising an alignment of 1238 nucleotides from the 16S ribosomal RNA genes of (with abbreviated names given in parentheses): *Aquifex pyrophilus* (Apyr), *Thermotoga maritima* (Tmar), *Thermus thermophilus* (Tthe), *Deinococcus radiodurans* (Drad), and *Bacillus subtilis* (Bsub). This alignment is similar to that analyzed by Galtier and Gouy (1995) and by Foster (2004).

#### 5.2.1 Assessment of phylogenetic assumptions

The matched-pairs tests of symmetry (Bowker 1948), marginal symmetry (Stuart 1955) and internal symmetry (Ababneh et al 2006b) show that the sequences are highly unlikely to have evolved under stationary and homogeneous conditions (Table 10). We analyzed this data set, in a similar manner to the hominoid data set, by (*i*) considering the maximum-likelihood results obtained using different models of DNA evolution; (*ii*) comparing the trees using the goodness-of-fit measure described in section 5.1.5; and (*iii*) comparing the trees based on tests of symmetry along individual edges. However, we did not perform the likelihood ratio test as the difference in log likelihood values obtained using the BH program and PAUP* is extremely large and could not have arisen by chance.

#### 5.2.2 Inferring the trees

**Figure 2:**
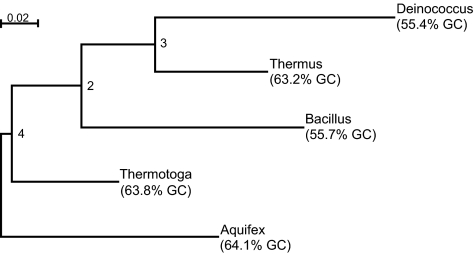
The most likely bacterial tree inferred by BH (tree #1). The GC content of the sequences is included (based on Table 14a).

**Figure 3:**
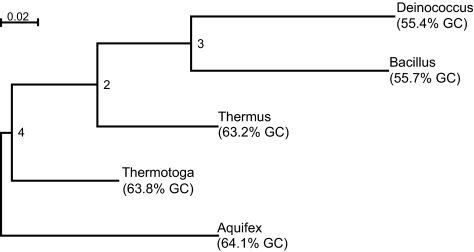
The most likely bacterial tree inferred using GTR (and F84) models (tree #2). The GC content of the sequences is included (based on Table 14a).

Using the BH program, log likelihood values were found to fall in the range from −4387.239 to −4289.511. The most likely tree obtained is shown in Figure 2 and henceforth referred to as tree #1. Like the other data set, the marginal probabilities at the internal nodes are consistent and the marginal probabilities at the leaf nodes fit the observed data precisely. The most likely tree inferred using DNAML and PAUP* is shown in Figure 3 and henceforth referred to as tree #2. Even the BH program returned this particular tree as the second most likely with a log likelihood value of −4296.22. By contrast, the log likelihood value returned by PAUP* for the same tree is −4401.41722. The large difference in log likelihood values returned by BH and PAUP* is expected because the evolutionary process is highly unlikely to have been stationary and homogeneous (Table 10) and, hence, cannot be approximated by the GTR model of DNA evolution. For the bacterial data set, Foster (2004) considered rooted trees with locally homogeneous processes acting on them and concluded that two different rate matrices were sufficient to describe the evolutionary process. However, Foster’s (2004) method requires the frequency parameters to be known in advance and he chose two sets of frequency parameters (and hence two different rate matrices) based on the observation that the marginal probabilites at leaf nodes could be grouped into two different categories. For a large tree, intrepreting the closeness of frequency parameters might prove to be a difficult task. Another possible limitation of Foster’s (2004) approach is that the change in rate matrices owing to changes in parameters other than the frequency parameters are not considered. For example, the GTR model has five free parameters in addition to the frequency parameters and a change in those five parameters would also change the rate matrix. This limitation exists even for the N1 and N2 models proposed by Yang and Roberts (1995). In contrast, the BH model uses the available data to automatically adjust the parameters from edge to edge and can be used to identify the portions of the tree where the rate matrix is homogeneous.

#### 5.2.3 Comparison of edge lengths

The edge lengths calculated using BH can be used to obtain the distance between a pair of leaf nodes. For example, the distance from *Thermus* to *Thermotoga* for tree #1 can be obtained by adding the distances along the edges (*Thermus, Node-3*), (*Node-3, Node2*), (*Node-2, Node-4*) and (*Node-4, Thermotoga*). For tree #1 we found that the distances between *Deino-coccus-Thermus* (0.191) and *Thermus-Thermotoga* (0.194) are quite close (Table 11a). Since the edge lengths are only an approximation, it is possible that phylogenetic programs that assume the process to be stationary, homogeneous and reversible would infer a tree favouring *Thermus* closer to the *Thermotoga-Aquifex* pair (tree #2), an observation supported by the output from DNAML and PAUP*, and by the fact that the five sequences can be divided into two groups according to their GC content, where the group containing GC-rich sequences comprises *Aquifex, Thermotoga* and *Thermus* and the other group comprises *Bacillus* and *Deinococcus* (Figure 3). Although the evolutionary process is not stationary and homogeneous for the bacterial data set, the edge lengths obtained using BH for tree #1 and tree #2 are within the confidence interval specified by DNAML (Table 11). The closeness of the edge lengths returned by these two programs for this data set shows that averaging of edge lengths might be considered an adequate approximation.

#### 5.2.4 Evaluation of Divergence Matrices and Substitution Biases

The closeness of estimated and observed divergence matrices can be measured using the goodness-of-fit index described in section 5.1.5. For tree #1, the estimated and observed divergence matrices are quite close to one another except for the pair *Bacillus-Deinococcus* (Table 12).

In contrast, the divergence matrices for tree #2 has a low goodness-of-fit index value for the *Bacillus-Deinococcus* pair but high indices for the *Bacillus-Thermotoga* and the *Bacillus-Aquifex pairs* (Table 13). Since significant differences exist between observed and estimated divergence matrices for both tree topologies, the evidence is insufficient to favour one particular tree over the other.

Since the marginal probabilities at the leaf nodes *Thermus*, *Thermotoga* and *Aquifex* and the internal nodes are quite close (Table 14a, Table 14b and Table 14c), and the marginal probabilities of *Bacillus* and *Deinococcus* (Table 14a) are close to one another, but different from those of the other taxa, tree #2 is the simplest model agreeing with the tests of symmetry. However, the BH program returned a slightly higher log likelihood value of −4289.511 for tree #1 compared to a value of −4296.22 for tree #2 and some authors (see eg Gupta 2000) have favoured the close relationship of *Thermus* and *Deinococcus* (as in tree #1).

We performed tests of symmetry along each edge for tree #1 and tree #2 using *NQ*(*x*,*y*), for *x*, *y* = 1, …, 4, as pseudo-observations (refer section 5.1.5) and the resulting pseudo-*p*-values are described in Table 15. As noted earlier, the evolutionary process is not stationary for the five-taxa tree and Table 15b suggests a stationary process for the sub-tree containing leaf nodes *Thermus*, *Thermotoga* and *Aquifex*, distinct from the process that gave rise to *Bacillus* and *Deinococcus*.

An examination of the estimated joint probabilities for edges in tree #1 shows that there are large biases in the patterns of substitutions along many of the edges. For example, in the case concerning the terminal edge to *Deinococcus*, there is a strong bias in A → C, A → G, T → C, and T → G substitutions over C → A, G → A, C → T, and G → T substitutions (Table 16).

## 6 Performance

We ran all programs on a dual processor 1.25 GHz PowerPC G4 with 512 MB of DDR SDRAM and Mac OS X version 10.3.9 as the operating system. For a five-taxa or seven-taxa tree with 1206 nucleotide sites per taxon, our program took approximately one second to compute the likelihood for a single tree. For a 10-taxa tree having 1202 sites per taxon, it took 4 seconds to compute the likelihood for a single tree. To compare the performance of BH program with PAUP* (Swofford 2002), we considered seven hominoid species (refer Section 5.1) and computed the likelihood of each of the 945 unrooted trees using the two programs. In PAUP* (Swofford 2002), we selected the GTR model (Lanave et al 1984) and determined the maximum-likelihood estimates of all the eight free parameters. We found that the BH program took 33% longer than PAUP* (Swofford 2002) to compute the likelihood of the 945 trees.

## 7 Conclusion

By modifying the GTR model such that it still has the constraints of stationarity and homogeneity but not reversibility, we can obtain the general 12parameter model, where the 12-paramaters correspond to elements in the rate matrix (excluding the stationary probabilities). An even more general model can be obtained by considering the 12-parameter model over each edge of the tree. The only assumption made by such a model is that each edge (*i*, *j*) has a Markovian process defined over it; such a general non-homogeneous model was proposed by Barry and Hartigan (1987a).

We have implemented the algorithm by Barry and Hartigan (1987a) and used it to analyse two data sets – a hominoid data set with apparent stationarity and homogeneity, and a bacterial data set with apparent violation of these assumptions. We have also compared the results obtained using two different approaches and found that if the tests of symmetry indicate the evolutionary process to be stationary and homogeneous, then the most likely trees inferred using the F84 model, the GTR model and the general non-homogeneous model are the same. However, the log likelihood values obtained under the GTR model differ significantly from the general non-homogeneous model, providing evidence that the evolutionary process violates some of the assumptions made by the GTR model. Although the assumptions of stationarity and homogeneity can be assessed using tests of symmetry, there is no test available for checking the reversibility condition. However, values of the joint probability distribution returned by the BH program can be examined for reversibility; a symmetric *Q*-matrix for edge (*i*, *j*) corresponds to a reversible process along that edge.

For a stationary and homogeneous process, the edge lengths obtained from the general nonhomogeneous model are within the confidence interval specified by the F84 model and, in this respect, there is no obvious gain in using the general non-homogeneous model for the hominoid data set.

If the process is not stationary and homogeneous, as in the case of bacterial data set, then the preferred tree obtained using the F84 and GTR models may differ from that obtained using the general nonhomogeneous model. The tests of symmetry using divergence matrices of leaf nodes favour the tree obtained using the F84/GTR model. However, these tests ignore the possibility of varying evolutionary rates along different edges of the tree. Similarly, the F84 and GTR models of nucleotide substitution assume a constant rate of substitution for the entire tree and are less likely to find instances of convergent evolution. In contrast, the general nonhomogeneous model incorporates rate heterogeneity along each edge and, therefore, is more likely to find instances of convergent evolution.

Finally, we have shown that the trees obtained using the general non-homogeneous model can be compared using a goodness-of-fit index that measures the closeness of expected and observed divergence matrices. For both the hominoid and bacterial data sets, the estimated joint probability distribution matrices were found to be asymmetric for some of the edges connecting internal nodes to lead nodes. This bias in substitution implies a lack of reversibility, so it may be inappropriate to analyse the sequences using phylogenetic models that assume stationary, homogeneous and reversible conditions.

## 8 Future work

Our implementation of Barry and Hartigan’s (1987a) algorithm assumes that the nucleotide sites are independent and identically distributed. However, to make their algorithm more general, we need to incorporate rate heterogeneity among sites. Felsenstein and Churchill (1996) proposed the inclusion of a hidden Markov model to allow for rate variations among sites; perhaps a similar model could be used in the context of the BH algorithm.

Secondly, our implementation of Barry and Hartigan’s (1987a) algorithm calculates the maximum-likelihood for a user-specified tree. It does not search the treespace for the most likely tree and therefore is limited to analysis of a small number of taxa (*k* ≤ 7). Although searching through the entire tree space is an NP-hard problem, the computation time can be reduced by using a search strategy such as branch and bound (Hendy and Penny 1982) or other heuristic methods. We have modified Lewis’ genetic algorithm (1998) to search through the tree space but the processing time is far greater than that required by PHYLIP (Felsenstein 2004a) or PAUP* (Swofford 2002). One possible way of reducing the processing time would be to implement a parallel version of the BH algorithm. Some of the other heuristic methods that might be useful are tree-fusing (Goloboff 1999) and simulated annealing (Metropolis et al 1953; Salter and Pearl 2001). We also need to search the likelihood surface more exhaustively and, if possible, identify the *Q*-values that converge to a global maximum. Finally, we need to understand better the statistical properties associated with assessment of symmetry of joint probability distribution matrices.

## Figures and Tables

**Table 1:** Probabilities obtained from matched-pairs tests of symmetry, marginal symmetry and internal symmetry using 1st codon sites from the hominoid data

		Ppan	Ptro	Hsap	Ggor	Ppyg	Hlar
**Ptro**	Bowker	0.206					
	Stuart	0.620					
	Ababneh	0.425					

**Hsap**	Bowker	0.217	0.709				
	Stuart	0.312	0.867				
	Ababneh	0.532	0.883				

**Ggor**	Bowker	0.032	0.219	0.302			
	Stuart	0.024	0.227	0.243			
	Ababneh	0.769	0.994	0.387			

**Ppyg**	Bowker	0.440	0.579	0.614	0.139		
	Stuart	0.092	0.095	0.239	0.078		
	Ababneh	1.000	1.000	1.000	0.680		

**Hlar**	Bowker	0.400	0.331	0.262	0.180	0.703	
	Stuart	0.517	0.419	0.576	0.106	0.696	
	Ababneh	0.268	0.404	0.127	0.688	0.499	

**Msyl**	Bowker	0.592	0.584	0.303	0.233	0.635	0.735
	Stuart	0.327	0.304	0.303	0.056	0.242	0.522
	Ababneh	0.759	0.786	0.313	0.914	0.989	0.913

**Table 2:** Probabilities obtained from matched-pairs tests of symmetry, marginal symmetry and internal symmetry using 2nd codon sites from the hominoid data

		Ppan	Ptro	Hsap	Ggor	Ppyg	Hlar
	Bowker	0.102					
**Ptro**	Stuart	0.206					
	Ababneh	1.000					

	Bowker	0.197	0.352				
**Hsap**	Stuart	0.348	0.826				
	Ababneh	1.000	0.754				

	Bowker	0.264	0.323	0.361			
**Ggor**	Stuart	0.437	0.706	0.334			
	Ababneh	1.000	0.352	0.558			

	Bowker	0.359	0.446	0.728	0.297		
**Ppyg**	Stuart	0.154	0.243	0.401	0.088		
	Ababneh	0.720	0.653	0.879	0.867		

	Bowker	0.157	0.444	0.126	0.331	0.165	
**Hlar**	Stuart	0.297	0.721	0.638	0.513	0.177	
	Ababneh	0.231	0.329	0.075	0.327	0.239	

	Bowker	0.710	0.957	0.890	0.605	0.46	0.801
**Msyl**	Stuart	0.881	0.996	0.940	0.940	0.494	0.948
	Ababneh	0.378	0.690	0.592	0.248	0.351	0.440

**Table 3:** Probabilities obtained from matched-pairs tests of symmetry, marginal symmetry and internal symmetry using 3rd codon sites from the hominoid data

		Ppan	Ptro	Hsap	Ggor	Ppyg	Hlar
	Bowker	0.670					
**Ptro**	Stuart	0.357					
	Ababneh	0.846					

	Bowker	0.517	0.504				
**Hsap**	Stuart	0.511	0.452				
	Ababneh	0.589	0.443				

	Bowker	0.257	0.767	0.171			
**Ggor**	Stuart	0.568	0.947	0.459			
	Ababneh	0.349	0.398	0.092			

	Bowker	0.019	0.028	0.016	0.046		
**Ppyg**	Stuart	0.016	0.029	0.242	0.011		
	Ababneh	0.180	0.160	0.010	0.662		

	Bowker	0.236	0.277	0.743	0.244	0.756	
**Hlar**	Stuart	0.083	0.135	0.623	0.093	0.535	
	Ababneh	0.715	0.584	0.627	0.678	0.748	

	Bowker	0.372	0.528	0.383	0.158	0.035	0.445
**Msyl**	Stuart	0.151	0.261	0.354	0.386	0.006	0.142
	Ababneh	0.996	0.986	0.567	0.100	0.811	0.948

**Table 4:** Log Likelihood values for the three most likely trees returned by the BH program

Tree	Log Likelihood
((((((Ptro,Ppan),Ggor),Hsap),Ppyg),Hlar),Msyl)	−3540.684
((((((Ptro,Ppan),Hsap),Ggor),Ppyg),Hlar),Msyl)	−3545.508
(((((Ptro,Ppan),(Hsap,Ggor)),Ppyg),Hlar),Msyl)	−3554.946

**Table 5:** Shimodaira-Hasegawa (SH) Test and Approximately Unbiased (AU) Test

Tree	SH Test	AU Test
((((((Ptro,Ppan),Ggor),Hsap),Ppyg),Hlar),Msyl)	0.811	0.716
((((((Ptro,Ppan),Hsap),Ggor),Ppyg),Hlar),Msyl)	0.428	0.334
(((((Ptro,Ppan),(Hsap,Ggor)),Ppyg),Hlar),Msyl)	0.075	0.026

**Table 6:** Comparison of edge lengths obtained using BH and PHYLIP for the hominoid tree ((((((Ptro,Ppan), Ggor), Hsap), Ppyg), Hlar), Msyl). Refer Figure 1 for an explanation of node numbers.

Edge	Distance using BH	Distance using DNAML	Confidence Interval (DNAML)
Ppyg, Node-2	0.058	0.061	0.046–0.077
Node-2, Node-4	0.028	0.024	0.014–0.035
Node-2, Node-3	0.018	0.020	0.011–0.030
Node-4, Hlar	0.037	0.039	0.027–0.053
Node-4, Msyl	0.108	0.109	0.088–0.129
Node-3, Hsap	0.032	0.029	0.019–0.040
Node-3, 5-Node	0.009	0.009	0.003–0.016
Node-5, Ggor	0.043	0.042	0.029–0.055
Node-5, Node-6	0.010	0.009	0.003–0.015
Node-6, Ptro	0.017	0.017	0.009–0.025
Node-6, Ppan	0.016	0.015	0.007–0.022

**Table 7:** Macaque-Bonobo divergence matrix for the seven taxa hominoid tree ((((((Ptro, Ppan), Ggor), Hsap), Ppyg), Hlar), Msyl) based on (a) observed values and (b) joint probability distribution values

**(a)**		**A**	**C**	**G**	**T**

	A	306	11	18	15
	C	10	279	2	47
	G	20	4	142	2
	T	6	40	2	302

**(b)**		**A**	**C**	**G**	**T**

	A	303.7	12.8	21.6	11.9
	C	10.2	270.8	2.1	54.8
	G	21.3	7.5	138.1	1.1
	T	6.8	42.8	2.2	298.2

**Table 8:** Probability values for Bowker’s Test of Symmetry and Stuart’s Test of Marginal Symmetry for all the edges of the most likely hominoid tree ((((((Ptro, Ppan), Ggor), Hsap), Ppyg), Hlar), Msyl). See Figure 1 for an explanation of node numbers

Edge	Bowker’s Test	Stuart’s Test
Ppyg, Node-2	0.113	0.035
Node-2, Node-4	0.435	0.697
Node-2, Node-3	0.241	0.282
Node-3, Hsap	0.000	0.000
Node-3, Node-5	0.145	0.023
Node-5, Ggor	0.001	0.000
Node-5, Node-6	0.088	0.012
Node-6, Ptro	0.085	0.013
Node-6, Ppan	0.097	0.013
Node-4, Hlar	0.454	0.140
Node-4, Msyl	0.135	0.080

**Table 9:** Contingency table for the edge linking node 5 to the Gorilla leaf node. Rows correspond to internal node and columns to leaf node

	A	C	G	T
A	325.0	2.0	17.7	3.0
C	2.0	332.4	0.0	18.5
G	2.0	0.0	156.3	0.0
T	0.0	4.6	0.0	342.5

**Table 10:** Probabilities obtained from matched-pairs tests of symmetry, marginal symmetry and internal symmetry using all sites from the bacterial data

		Apyr	Bsub	Drad	Tthe
	Bowker	0.000			
**Bsub**	Stuart	0.000			
	Ababneh	0.295			

	Bowker	0.000	0.995		
**Drad**	Stuart	0.000	0.946		
	Ababneh	0.754	0.958		

	Bowker	0.509	0.000	0.000	
**Tthe**	Stuart	0.731	0.000	0.000	
	Ababneh	0.263	0.544	0.863	

	Bowker	0.132	0.000	0.000	0.415
**Tmar**	Stuart	0.325	0.000	0.000	0.267
	Ababneh	0.095	0.417	0.297	0.546

**Table 11 (a):** Comparison of edge lengths obtained using the BH program and DNAML for tree #1. Refer Figure 2 for tree diagram and an explanation of node numbers

Edge	Distance using BH	Distance using DNAML	Confidence Interval (DNAML)
Bsub, Node-2	0.122	0.127	0.104–0.150
Node-2, Node-3	0.040	0.039	0.024–0.053
Node-3, Tthe	0.060	0.069	0.051–0.087
Node-3, Drad	0.131	0.120	0.098–0.143
Node-2, Node-4	0.036	0.043	0.027–0.058
Node-4, Tmar	0.058	0.061	0.044–0.078
Node-4, Apyr	0.124	0.127	0.104–0.150

**Table 11 (b):** Comparison of edge lengths obtained using the BH program and DNAML for tree #2. Refer Figure 3 for tree diagram and an explanation of node numbers

Edge	Distance using BH	Distance using DNAML	Confidence Interval (DNAML)
Tthe, Node-2	0.064	0.068	0.050–0.086
Node-2, Node-3	0.050	0.050	0.033–0.066
Node-3, Bsub	0.106	0.105	0.083–0.126
Node-3, Drad	0.110	0.108	0.087–0.130
Node-2, Node-4	0.046	0.047	0.031–0.063
Node-4, Tmar	0.059	0.063	0.046–0.079
Node-4, Apyr	0.122	0.122	0.099–0.145

**Table 12:** Divergence matrices for tree #1 for (a) Bacillus-Aquifex pair and (b) Bacillus-Deinococcus pair

(a) Observed and estimated divergence matrix values for *Bacillus-Aquifex* pair										
**(i)**		**A**	**C**	**G**	**T**	**(ii)**	**A**	**C**	**G**	**T**

	A	0.195	0.019	0.034	0.004		0.191	0.018	0.039	0.004
	C	0.005	0.201	0.02	0.012		0.006	0.194	0.024	0.014
	G	0.012	0.030	0.273	0.004		0.014	0.038	0.262	0.005
	T	0.002	0.037	0.027	0.125		0.003	0.037	0.03	0.121
(b) Observed and estimated divergence matrix values for *Bacillus-Deinococcus* pair										
**(i)**		**A**	**C**	**G**	**T**	**(ii)**	**A**	**C**	**G**	**T**

	A	0.209	0.007	0.023	0.011		0.199	0.012	0.032	0.008
	C	0.006	0.192	0.017	0.023		0.012	0.176	0.019	0.031
	G	0.023	0.015	0.271	0.011		0.032	0.018	0.252	0.017
	T	0.012	0.019	0.011	0.149		0.008	0.027	0.017	0.139

**Table 13:** Goodness of Fit index for all pairs of bacteria

Sequence Pair	Tree #1	Tree #2
Bsub-Tmar	3.06	17.94
Bsub-Apyr	7.01	25.37
Bsub-Tthe	1.26	0.91
Bsub-Drad	34.92	3.41
Tmar-Apyr	0.52	0.43
Tthe-Drad	2.10	13.65
Tmar-Drad	3.14	4.59
Apyr-Drad	5.99	6.85
Tmar-Tthe	7.90	1.42
Apyr-Tthe	9.06	1.77

**Table 14:** 

(a) Marginal probabilities at leaf nodes for bacterial data set.				
**Leaf Node**	**A**	**C**	**G**	**T**

Tthe	0.219	0.278	0.354	0.149
Tmar	0.207	0.279	0.359	0.155
Apyr	0.214	0.287	0.354	0.145
Drad	0.250	0.233	0.321	0.195
Bsub	0.251	0.238	0.319	0.191
(b) Marginal probabilities at internal nodes for tree #1.				
**Internal Node**	**A**	**C**	**G**	**T**

Node-2	0.216	0.272	0.358	0.154
Node-3	0.218	0.269	0.357	0.156
Node-4	0.210	0.282	0.36	0.148
(c) Marginal probabilities atinternal nodes for tree #2.				

**Internal Node**	**A**	**C**	**G**	**T**

Node-2	0.214	0.275	0.360	0.151
Node-3	0.227	0.257	0.342	0.174
Node-4	0.212	0.281	0.361	0.146

**Table 15:** Probability values for Bowker’s Test of Symmetry and Stuart’s Test of Homogeneity for all the edges of the bacterial tree

(a) Tree #1. Refer Figure 2 for an explanation of node numbers		
**Edge**	**Bowker’s Test**	**Stuart’s Test**

Bsub, Node-2	0.000	0.000
Node-2, Node-3	0.427	0.835
Node-2, Node-4	0.002	0.005
Node-3, Tthe	0.567	0.390
Node-3, Drad	0.000	0.000
Node-4, Tmar	0.646	0.359
Node-4, Apyr	0.135	0.742
(b) Tree #2. Refer Figure 3 for an explanation of node numbers		
**Edge**	**Bowker’s Test**	**Stuart’s Test**

Tthe, Node-2	0.568	0.607
Node-2, Node-3	0.000	0.000
Node-2, Node-4	0.167	0.264
Node-3, Drad	0.000	0.000
Node-3, Bsub	0.000	0.000
Node-4, Tmar	0.315	0.092
Node-4, Apyr	0.130	0.843

**Table 16:** Contingency table for the edge linking node 3 to the Deinococcus leaf node. Rows correspond to internal node and columns to leaf node.

a	A	C	G	T
**A**	260.2	12.8	37.9	0.0
**C**	2.1	280.7	6.2	6.0
**G**	4.7	9.7	378.0	2.6
**T**	0.5	32.9	21.2	182.3
